# Evaluation of a five-year predicted survival model for cystic fibrosis in later time periods

**DOI:** 10.1038/s41598-020-63590-8

**Published:** 2020-04-20

**Authors:** Theodore G. Liou, Christiana Kartsonaki, Ruth H. Keogh, Frederick R. Adler

**Affiliations:** 1 Center for Quantitative Biology, University of Utah, Salt Lake City, Utah USA; 2 The Adult Cystic Fibrosis Center at the University of Utah, Division of Respiratory, Critical Care and Occupational Pulmonary Medicine, Department of Internal Medicine, School of Medicine, University of Utah, Salt Lake City, Utah USA; 3 Clinical Trial Service Unit & Epidemiological Studies Unit and Medical Research Council Population Health Research Unit, Nuffield Department of Population Health, University of Oxford, Oxford, United Kingdom; 4 Department of Medical Statistics, London School of Hygiene and Tropical Medicine, London, United Kingdom; 5 Department of Mathematics, College of Science and the College of Biological Sciences, University of Utah, Salt Lake City, Utah USA

**Keywords:** Cystic fibrosis, Epidemiology, Risk factors, Infection, Diabetes

## Abstract

We evaluated a multivariable logistic regression model predicting 5-year survival derived from a 1993–1997 cohort from the United States Cystic Fibrosis (CF) Foundation Patient Registry to assess whether therapies introduced since 1993 have altered applicability in cohorts, non-overlapping in time, from 1993–1998, 1999–2004, 2005–2010 and 2011–2016. We applied Kaplan-Meier statistics to assess unadjusted survival. We tested logistic regression model discrimination using the C-index and calibration using Hosmer-Lemeshow tests to examine original model performance and guide updating as needed. Kaplan-Meier age-adjusted 5-year probability of death in the CF population decreased substantially during 1993–2016. Patients in successive cohorts were generally healthier at entry, with higher average age, weight and lung function and fewer pulmonary exacerbations annually. CF-related diabetes prevalence, however, steadily increased. Newly derived multivariable logistic regression models for 5-year survival in new cohorts had similar estimated coefficients to the originals. The original model exhibited excellent calibration and discrimination when applied to later cohorts despite improved survival and remains useful for predicting 5-year survival. All models may be used to stratify patients for new studies, and the original coefficients may be useful as a baseline to search for additional but rare events that affect survival in CF.

## Introduction

Survival of patients with cystic fibrosis (CF) has improved worldwide^1–6^. Improved care over the last 80 years increased the expectation of life at birth from a median of six months to approaching 50 years^2,7,8^. Patients, their families, friends and caretakers often compare individual age at death with the expectation of life at birth as a final measure of their efforts to extend a single life. However, this comparison is overly stringent and cannot account for individual circumstances that markedly change outcomes. If truly desired, one might look instead to the aggregated median age at death which reflects the age-distribution of the contemporary CF population, current mortality rates and the improving conditional survival from all ages as a result of rapidly improving therapies^3,9^. Recently, this measurement rose to nearly 30 years^2^. Currently recommended^10–13^ beneficial treatments include pancreatic enzymes^10,14^, airway clearance^15,16^, mucolysis^17–19^, inhaled antibiotics^20–22^, anti-inflammatory agents^23,24^, and CF transmembrane regulator protein (CFTR) modulators^25–27^. Although gains have been tremendous, CF survival continues to fall short of the approximately 80 year expectation of life for the average newborn in the United States^28,29^, providing continued motivation to improve treatments^2^.

To help clinicians better understand relative survival associations of different demographic and disease-related factors, we previously developed and tested a 5-year predictive survivorship model for CF^30^ using a cohort of patients alive and enrolled on January 1, 1993 in the US CF Foundation Patient Registry (CFFPR) followed through 1997 (Table 1). Because part of the original intent was to create a clinically useful tool, we chose logistic regression which most easily produces a single risk assessment derived from multiple but readily obtained measurements at the point of care.

**Table 1:** Multivariable Logistic Regression 5-Year Predicted Survival Model, US CFFPR, Originally Published 1993–1997 Cohort^*^.

Variables	Parameter Estimates (log odds ratios)	Standard Errors	Odds Ratio (95% Confidence Interval)	*P* value
Intercept	−1.93	0.27	0.14 (0.08–0.25)	< 0.001
Age (per year)	0.028	0.0060	1.03 (1.02–1.04)	< 0.001
Sex (male = 0, female = 1)	0.23	0.10	1.26 (1.04–1.53)	0.018
FEV_1_% (per %)	−0.038	0.0028	0.96 (0.96–0.97)	< 0.001
Weight-for-age *z* Score	−0.40	0.053	0.67 (0.6–0.74)	< 0.001
Pancreatic sufficiency (0 or 1)	−0.45	0.31	0.64 (0.35–1.16)	0.141
Diabetes mellitus (0 or 1)	0.49	0.15	1.63 (1.21–2.21)	0.001
Methicillin sensitive *Staphylococcus aureus* (0 or 1)	−0.21	0.12	0.81 (0.64–1.02)	0.067
*Burkholderia cepacia* (0 or 1)	1.82	0.30	6.20 (3.42–11.23)	< 0.001
Number of prior year acute exacerbations (0–5)	0.46	0.031	1.59 (1.5–1.69)	< 0.001
Interaction between prior year acute exacerbations and *B. cepacia*	−0.40	0.12	0.67 (0.53–0.84)	0.001

^*^Reproduced with permission and modified by reversal of sign of parameter estimates to predict deaths rather than survival and addition of 95% confidence intervals with *P* values^30^.

The most important single variable is the forced expiratory volume in 1 second (FEV_1_), a measurement of airway physiology, expressed as the percent predicted FEV_1_ (FEV_1_%) estimated from each patient’s age, sex, height, race and ethnicity^31,32^. Every percentage point higher value of FEV_1_% indicates a 4% reduction in 5-year risk of death on average, all other factors being equal (odds ratio, OR = 0.96, 95% Confidence Interval [CI] 0.957–0.968, *P* < 0.001). *Burkholderia cepacia* has the highest impact of any single factor implying that the infection increases the risk of death more than six-fold (OR = 6.20, 95% CI 3.42–11.23, *P* < 0.001). Fortunately, the number of patients with this infection was small in 1993 (and remains small, Table 2). The size of the effect of *B cepacia* is partially explained because of an interaction with the number of pulmonary exacerbations in the year prior to evaluation. Each observed pulmonary exacerbation signals a 59% increased risk of death within 5 years (OR = 1.59 per exacerbation, 95% CI 1.50–1.69, *P* < 0.001). The interaction term indicates that pulmonary exacerbations have a smaller expected survival effect in the presence of *B cepacia* infection. Thus, exacerbations in patients with *B cepacia* infection have an effect that is reduced by about a third by the interaction term (OR = 0.67, 95% CI 0.53–0.84, *P* = 0.001) compared to exacerbations in the absence of that infection. A diagnosis of CF-related diabetes (CFRD) has the same effect on 5-year predicted survival as a 12 percentage point reduction in FEV_1_% (OR = 1.63, 95% CI 1.21–2.21, *P* = 0.001). The finding quantified the high impact of the diagnosis in CF^33,34^. Two variables were included in the original multivariable model despite high *P* values, *Staphylococcus aureus* infection (OR = 0.81, 95% CI 0.64–1.02, *P* = 0.07) and pancreatic sufficiency (OR = 0.635, 95% CI 0.35–1.16, *P* = 0.14) for two reasons. First, they are prominent features of the clinical syndrome that are useful signals of health and disease as commonly assessed in isolation at the bedside, and, second, these variables substantially improved the fit of the overall model to the data.

**Table 2:** Baseline Characteristics of US CFFPR Cohorts, 1993–2016.

		Original 1993–1997 Cohort, n = 11,630^30^	New 1993–1997 Cohort, n = 9,941	New 5-Year Cohorts^*^			
				1993–1998, n = 9,757	1999–2004, n = 13,073	2005–2010, n = 15,043	2011–2016, n = 17,635
Deaths within 5 years: %		12.7	12.2 ^†^	13.2 ^†^	10.0 ^‡^	7.5 ^‡^	7.3 ^‡^
Age: Median (Range)		15.42 (5.50–71.05)	15.37 (6.00–71.05) ^†^	15.44 (6.00–66.47) ^†^	15.29 (6.01–72.15) ^†^	16.25 (6.00–74.31) ^§^	17.93 (6.00–81.14) ^§^
Sex: % Female		46.6	46.8 ^†^	46.8 ^†^	47.0 ^†^	47.6 ^†^	48.5 ^ǁ^
FEV_1_%: Median (Range)		67.94 (5.11–191.46)	70.92 (6.02–184.13) ^**^	74.52 (6.02–184.13) ^**^	85.79 (9.52–174.36) ^**^	88.49 (14.57–178.99) ^**^	91.43 (11.00–196.24) ^**^
Weight-for-age *z*-score: Mean (SD)		−0.85 (1.06)	−0.83 (1.05) ^†^	−0.76 (1.05) ^§^	−0.46 (1.00) ^§^	−0.25 (1.01) ^§^	−0.08 (1.00) ^§^
Pancreatic Sufficiency: % Affected		5.3	6.3 ^††^	4.2 ^‡^	4.5 ^††^	6.1 ^††^	9.7 ^‡^
Diabetes: % Affected		6.2	6.3 ^†^	7.0 ^§§^	9.5 ^‡^	14.6 ^‡^	21.1 ^‡^
Methicillin Sensitive *S aureus*: % Infected		30.8	30.9 ^†^	36.3 ^‡^	48.6 ^‡^	61.1 ^‡^	61.3 ^‡^
*B cepacia* complex: % Infected		3.6	3.7 ^†^	4.0 ^†^	3.9 ^†^	3.7 ^†^	3.9 ^†^
Prior Year Pulmonary Exacerbations: Median (Range)		0 (0–5)	0 (0–5) ^‡^	0 (0–5) ^‡^	0 (0–5) ^‡^	0 (0–5) ^‡^	0 (0–5) ^‡^
% with Number of Pulmonary Exacerbations^‖‖^	0	51.7	59.6	59.3	66.5	59.5	59
	1	19.5	17.3	17.5	11.7	23.2	22.3
	2	12.8	10.8	10.8	10.2	9.2	10.4
	3	6.8	5.5	5.5	4	4	4.3
	4	3.9	3.3	3.3	3	2.2	1.9
	5+	5.3	3.6	3.6	4.6	2	2.2
Prognostic risk score: Mean (SD)^***^		−3.10 (1.94)	−3.30 (1.83)	−3.43 (1.84)	−3.98 (1.81)	−4.18 (1.69)	−4.26 (1.73)
Prognostic risk score: Median (Range)^***^		−3.29 (−8.89–2.71)	−3.51 (−8.83–2.37)	−3.65 (−8.83–2.07)	−4.28 (−9.57–2.12)	−4.43 (−10.71–1.64)	−4.50 (−10.16–1.79)
100 × Predicted Probability of Death within 5 Years Using the Original Model: Median (Range)		3.597 (0.014–93.76)	2.902 (0.015–91.45)	2.535 (0.015–88.77)	1.359 (0.007–89.27)	1.177 (0.002–83.75)	1.098 (0.004–85.68)

^*^Comparisons used corrected data for FEV1, height and weight and weight-for -age *z*-score, prognostic risk score and predicted probabilities of death calculated using those corrected values.

Compared to original 1993–1997 cohort ^†^*P*-value not significant; ^‡^*P* < 0.001 by χ^2^; ^§^*P* < 0.001 by *t*-test; ^‖^*P* = 0.001 by χ^2^; ^**^P < 0.001 by Mann-Whitney-Wilcoxon test; ^††^*P* < 0.01 by χ2; ^§§^*P* < 0.05 by χ^2^.

^‖‖^Statistical comparisons were not made for percentages of patients with each Number of Pulmonary Exacerbations. See main text paragraph in Results concerning prognostic risk scores.

^***^Prognostic risk score is the equivalent to the log-odds of death within 5-years.

Clinicians and researchers currently use the model to understand survival implications. For example, Rubin *et al*.^29^ projected long-term survival outcomes of CFTR modulator use for patients homozygous for F508del mutations using the original 5-year predicted model using the coefficients from the proportional hazards version of the model^30^.

Since 1993, death rates with CF have dramatically decreased^2^, the majority of effective CF-specific therapies were introduced^17,18,20,21,24–27^, and the CFFPR itself was extensively edited to improve data quality and comply with current privacy and data use practices^1^. Considering these changes, we examined shifts in the distributions of factors underlying 5-year survival and assessed prediction model usefulness. The original model incorporated commonly measured and recorded clinically important variables on which clinicians continue to focus. Thus, we evaluated the original variables in CFFPR-derived patient cohorts from later time periods, examined model discrimination and calibration and considered the need for modifications.

## Methods

### Data

This study was performed in accordance with Good Clinical Practice and the Declaration of Helsinki. The University of Utah Investigational Review Board (IRB) assessed the ethics of our procedures and approved our study. After review, we requested the CFFPR 1986–2015 supplemented by 2016 outcomes data from the US CF Foundation (Bethesda, MD, USA). CFFPR data are collected from patients or their guardians after written informed assent (if 12–18 years old) and consent. All data are acquired with local IRB approval in accredited US CF care centers and affiliate programs^2^.

### Study design

To assess original model performance^30^, we selected patients from the CFFPR who were alive and seen at least once in 1993, 1999, 2005 or 2011 to create new cohorts to compare to the original cohort. Using the original patient selection criteria^30^, we created a new 1993–1997 cohort with January 1, 1993 as the time origin with follow up until December 31, 1997 or death for comparison with the original cohort to better understand data cleaning effects^1^. Patients without death dates were censored on December 31, 1997.

We used first encounter dates during 1993, 1999, 2005 or 2011 as the cohort time origins for 1993–1998, 1999–2004, 2005–2010 and 2011–2016, respectively, with follow up to death or last encounter within five years of the entry date. Loss to follow up was defined as having a final recorded contact with a patient before the end of 5 years of follow up for any study cohort without a record of death. These individuals were included as members of the group who remain alive at the end of each 5-year cohort study period. To address potential impact of loss to follow up during each cohort period, we treated censored patients as having died in sensitivity analyses. The 1993–1998 cohort allows assessment of using actual encounter dates for study inclusion compared to the original 1993–1997 cohort^30^.

The 5-year survival model^30^ includes nine variables, of which some require calculation from underlying variables (Table 1). For inclusion in a cohort, patients had to be at least 6 years old at one or more clinic encounters in the first year of each cohort, 1993, 1999, 2005 or 2011. At the baseline encounter for each cohort, patients needed height, weight and FEV_1_ measurements, pulmonary exacerbation counts for the prior year, pancreatic sufficiency status, CFRD status and sputum culture data including methicillin-sensitive *Staphylococcus aureus* (MSSA) and *Burkholderia cepacia* complex infections. Repeating our prior method^30^, we excluded patients if they received lung transplantation during or prior to each cohort period for the main analysis and had no other specific exclusion criteria. We repeated the entire analysis to understand the sensitivity to inclusion of patients who underwent lung transplantation during each cohort period.

### Initial data processing

We applied National Health and Nutrition Examination Survey (NHANES) III equations^31^ to the best FEV_1_ upon or in the year prior to cohort entry to derive FEV_1_% as these were the values primarily used during the periods of study and in the previous publication^30^. We used more recent Global Lung Initiative (GLI) equations^32^ to re-calculate FEV_1_% to assess the potential impact on results and interpretations due to the potential change in patients studied due to the differing availability of equations for specific ethnic and racial backgrounds. We used patient age on the date of start of follow up and the highest FEV_1_, worst microbial culture results and count of pulmonary exacerbations in the year prior to the start of follow up. We used reported insulin and pancreatic enzyme treatments as indicators of CFRD and pancreatic sufficiency, respectively. Weight-for-age *z*-score was calculated as done previously^30,35,36^.

We developed a method to identify and correct weight, height and FEV_1_ values that appeared incorrect for reasons such as recording values in incorrect units (for example, 182 cm measured as 72 inches and reported as 72 cm) or entering wrong values (for example, misrecorded digits such as 182 cm recorded as 82 cm or as 128 cm). These types of mistakes are usually identifiable with review of patient-specific longitudinal data but are often within physiologically normal ranges (Fig. S1). To standardize the process, we fitted generalized additive models (GAM)^37,38^ to identify weight, height and FEV_1_ values unlikely to be correct. For each patient, we treated height, weight and FEV_1_ as dependent variables and age as the independent variable. For each fit, we computed the standard deviation of the residuals and used this to find the *z*-score for the residual for each individual data point. We removed values with absolute *z*-scores greater than 3 for height and 4 for weight. We repeated the fits and removal of values with absolute *z*-scores greater than 3 or 4 on each successive fitting until no outstanding values remained. For FEV_1_, we did not correct values with negative *z*-scores because acute decreases are expected with pulmonary exacerbations. We used a *z*-score cutoff of 4 to identify high values likely to be incorrect without falsely identifying similar values following lung transplantation. We used the final individual GAM fits to estimate replacement values for height, weight and FEV_1_ values flagged as incorrect. However, we did not correct first or last values to avoid extrapolation outside the bounds of measured data. The main analysis used data from patients with complete data sets after corrections, but we repeated the analyses three times to understand model sensitivity to using complete data sets with (1) uncorrected but physiologically plausible, (2) corrected or (3) corrected and imputed data.

### Statistical analysis

We calculated Kaplan-Meier 5-year death rates for the four new cohorts, using each patient’s first encounter with complete data as the time origin. We summarized each predictor variable included in the original model and tested for differences between the original 1993–1997 cohort and all other cohorts. We examined each disease characteristic in the whole CFFPR by year to understand whether changes in distribution of values were isolated to study cohorts only^39,40^. We further evaluated year-to-year changes in CFRD prevalence using a multivariable model using generalized estimating equations with an independence working correlation matrix with CFRD as the outcome variable and age as the input variable adjusted by FEV_1_%, weight-for-age *z*-score and CFFPR year of study^41,42^.

We assessed model discrimination and calibration^43,44^. We used the original model^30^ to calculate a prognostic risk score in the new cohorts defined for individual $i$ as

1 $${p}_{i}={b}_{0}+{b}_{1}{x}_{{1}_{i}}+{b}_{2}{x}_{{2}_{i}}+\cdots +{b}_{10}{x}_{{10}_{i}},$$

where ${x}_{{1}_{i}},\ldots ,{x}_{{10}_{i}}$ denote the values for individual *i* of the variables included in the original model (including the interaction term, Table 1), ${b}_{0}$ is the model intercept, and *b*_1_, …, *b*_10_ are the estimated parameters (log odds ratios) from the original model corresponding to each variable. For patient, *i*, the predicted probability of death during 5-year follow-up is given by $P{R}_{i}=\frac{{e}^{{p}_{i}}}{1+{e}^{{p}_{i}}}$. We compared the distribution of the prognostic risk score across the new cohorts. To assess model discrimination, we derived the area under the receiver operating characteristic (ROC) curve or C-index^45,46^.

To assess model calibration, we divided subjects for each study cohort into 10 sub-groups, indexed by $g$, based on predicted probabilities of death $P{R}_{i}$(0–0.1, 0.1–0.2,…,0.9–1) and calculated the expected number of deaths within each sub-group during follow-up, ${E}_{g}={\sum }_{i\in g}P{R}_{i}$, and compared to the observed number of deaths both graphically and using a χ-squared test with 9 degrees of freedom^47,48^. To further assess model calibration, we fitted a logistic regression model in each cohort, with the indicator of 5-year mortality as the outcome for each patient, *Y*_*i*_, and the prognostic risk score as the predictor:

2 $$log\frac{Pr\,({Y}_{i}=1)}{1-Pr\,({Y}_{i}=1)}={\alpha }_{0}+{\alpha }_{1}\,{p}_{{\rm{i}}}.$$

In a perfectly calibrated model, the intercept (${\alpha }_{0}$) and slope (${\alpha }_{1}$) from the regression should be 0 and 1, respectively^44,49^. We considered two approaches^50^ to further assess and improve the performance of the original model using the original 9 covariables and 1 interaction term for the five new cohorts:

(1) The calibration intercept method allows a different intercept for the prognostic model in each new cohort, by changing the value of ${b}_{0}$ in Eq. 1. The new intercept is $\hat{\alpha }+{b}_{0}$ obtained by fitting model (2) with the slope (${\alpha }_{1}$) fixed at 1. An estimate with ${\hat{\alpha }}_{0} < 0$ indicates that the predicted probabilities obtained from the model are systematically too high while ${\hat{\alpha }}_{0} > 0$ indicates they are too low. The modified prognostic risk score is:

3 $${p}_{i}^{(1)}={\hat{\alpha }}_{0}+{b}_{0}+{b}_{1}{x}_{{1}_{i}}+{b}_{2}{x}_{{2}_{i}}+\cdots +{b}_{10}{x}_{{10}_{i}}.$$

(2) The calibration intercept and slope method systematically alters parameters *b*_1_, …, *b*_10_ in Eq. (1) by a constant multiplicative factor and derives a new intercept in place of ${b}_{0}$. The calibration intercept and slope are obtained by fitting model (2), and the prognostic risk score is:

4 $${p}_{i}^{(2)}={\hat{\alpha }}_{0}+{\hat{\alpha }}_{1}({b}_{0}+{b}_{1}{x}_{{1}_{i}}+{b}_{2}{x}_{{2}_{i}}+\cdots +{b}_{10}{x}_{{10}_{i}}).$$

Under each method we compared expected and observed probabilities of 5-year survival in 10 risk sub-groups as described above^47^.

We used the statistical system R to create study cohorts and perform all analyses^51^.

## Results

The CFFPR 1986–2016 contains data from 307 US CF Foundation accredited care center programs on 48,976 patients reported annually for 1986–1993, quarterly for 1994–2002 and with each of 1,771,761 clinical encounters for 2003–2015. Among these patients, 29,251 met inclusion criteria for one or more of the study cohorts formed in 1993, 1999, 2005 and 2011 (Table 2). Application of GLI^32^ rather than NHANES III equations^31^ on directly measured forced expiratory volume in one second (FEV_1_) values to derived percent predicted FEV_1_ (FEV_1_%) did not substantially change the patterns of inclusions or exclusions. The primary reasons for exclusion were age less than 6 years, missing data or lung transplantation (Table S1). Missingness increased with identification of incorrectly recorded values for height, weight and FEV_1_, but sensitivity analyses using uncorrected, corrected, or corrected and imputed data produced no evidence that data were not missing completely at random. Further sensitivity testing by inclusion of patients undergoing lung transplantation during each study cohort period had no substantial effect on results and no effect on interpretations.

The new 1993–1997 cohort has few changed characteristics relative to the original published 1993–1997 cohort. We found a small clinically unimportant increase in FEV_1_% values, more frequent pancreatic sufficiency and fewer pulmonary exacerbations (Table 2) suggesting that data cleaning since the original analysis^1^ does not substantially affect the applicability of the original model publication^30^.

**Figure 1:**
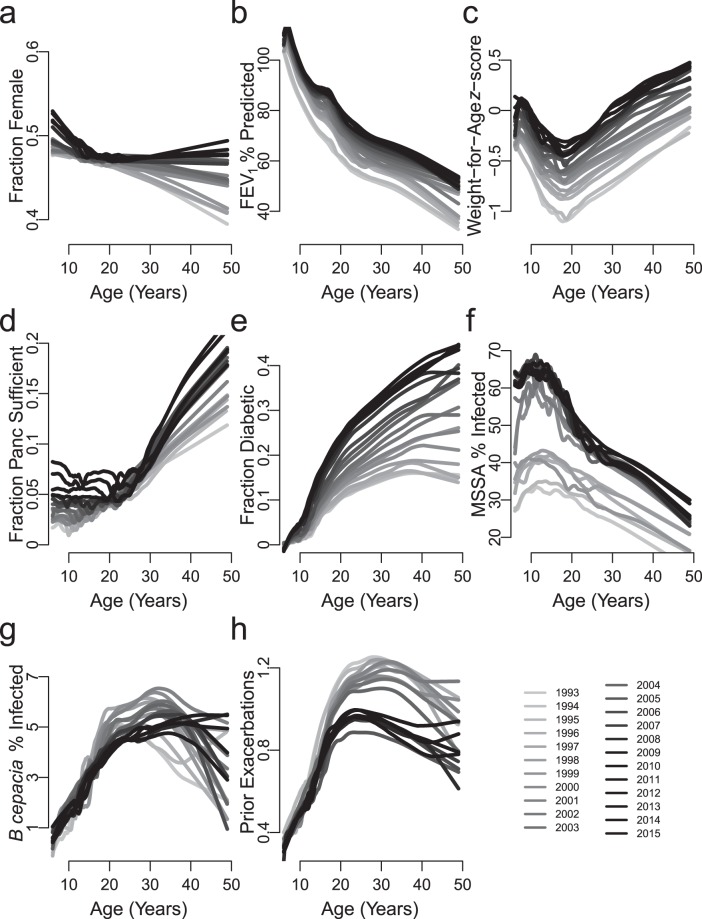
Prevalence of Conditions Predictive of 5-Year Survival in the US CFFPR, 1993–2015. Variables included in the original 5-year survival prediction model were evaluated by Registry year through 2015 for every age. (**A**) Sex distribution, (**B**) FEV_1_%, (**C**) Weight-for-Age *z*-score, (**D**) Pancreatic Sufficiency status, (**F**) Methicillin sensitive *S aureus* (MSSA) infection status, (**G**) *B cepacia* complex infection status, (**H**) Number of Pulmonary Exacerbations in the Prior Year all changed in directions consistent with improved long term survival. Of the variables in the original model, only (**E**) CF-Related Diabetes status worsened.

However, the distributions of patient characteristics for the more recent and new 5-year cohorts differ from the original 1993–1997 cohort. Most changes reflect improving trends in the CFFPR (Fig. 1A–D, F–H). In contrast, CF-related *Diabetes mellitus* (CFRD) prevalence as a function of age worsened (Fig. 1E) by about 9% per year (*P* <0.001, Table S2), a finding unexplained in multivariable analysis by increasing FEV_1_% (Fig. 1B) or weight-for-age *z*-score (Fig. 1C), which are negatively^52^ and independently associated (Table S2) and incompletely explained by modestly improving detection^34^.

**Figure 2:**
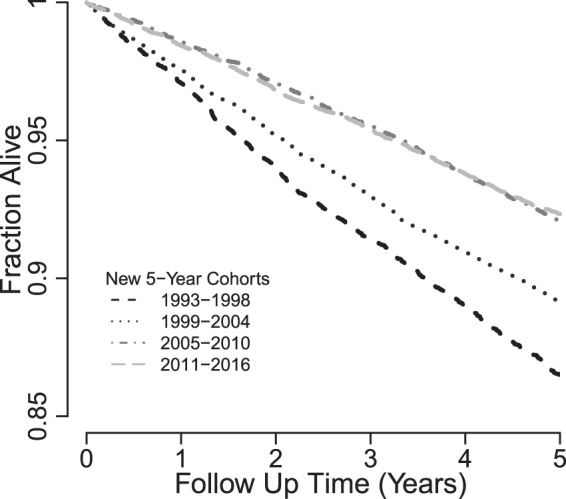
Unadjusted Kaplan-Meier Survivor Curves, US CFFPR, 1993–2016.

**Figure 3:**
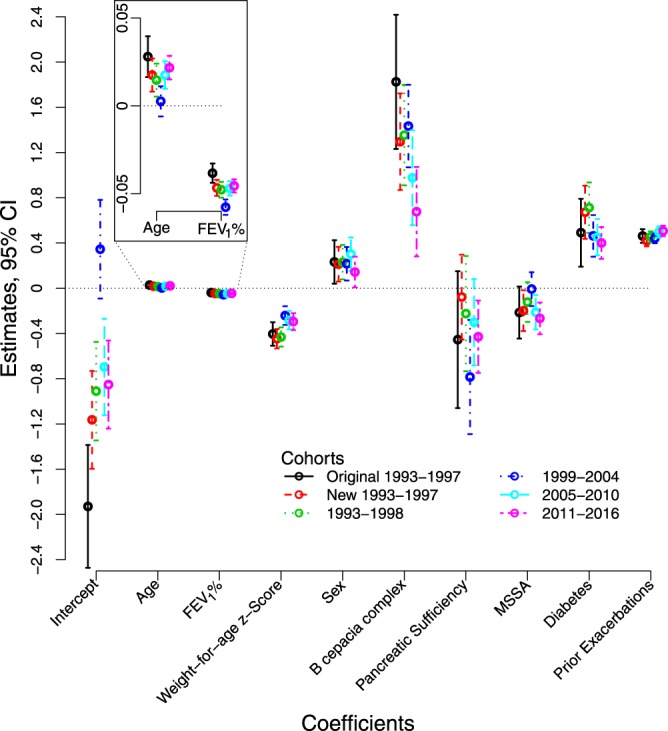
Coefficients from Re-Derivation of 5-Year Multivariable Logistic Regression Models by Cohort from the US CFFPR, 1993–2016. Coefficients with 95% confidence intervals are shown as derived from applying multivariable logistic regression to the cohorts studied: original 1993–1997 (derivation and validation cohorts combined)^30^, new 1993–1997, 1993–1998, 1999–2004, 2005–2010, 2011–2016.

**Figure 4:**
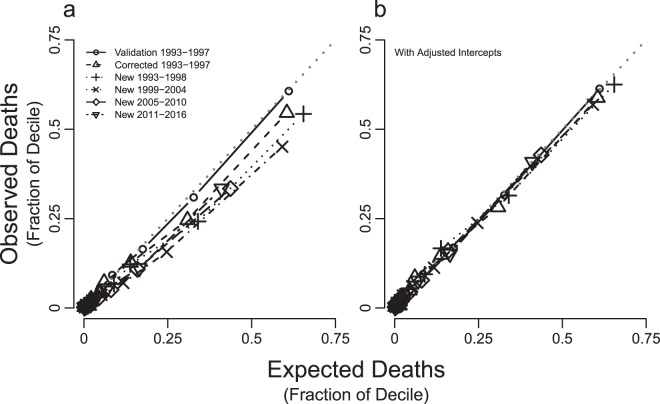
Comparisons of Observed and Expected Deaths for Studied Cohorts Using the Original 5-Year Predicted Survival Model, US CFFPR, 1993–2016. Observed and Expected deaths were derived by creating deciles of patients for Hosmer-Lemeshow testing. The fractions of deaths within each decile sub-group are plotted. (**A**) shows the discrimination performance of the original 5-year predicted survival applied to each of the cohorts studied in this work. (**B**) shows the effect of modifications of intercepts for each cohort.

The distribution and range of individual prognostic risk scores (log-odds ratios for death within 5-years) derived using the original 5-year predicted survival model (Table 1)^30^ were similar between all new cohorts and the original 1993–1997 cohort (Table 2). There was no significant difference between the prognostic risk scores from the new 1993–1997 cohort relative to those from the original. Scores for successive cohorts tended to be lower on average. Estimated Kaplan-Meier 5-year death probabilities decreased with successive study cohorts (Fig. 2), although without adjustment, differences could partially be due to age distribution changes at the start of each cohort. Nevertheless, histograms of prognostic risk scores and predicted probabilities showed similar distributions with no obvious outliers (Fig. S2). Refitting the 5-year multivariable logistic regression prediction model produced new model coefficients similar to published coefficients (Fig. 3 and Table S3), with the exception of the intercepts indicating that associations between predictors and outcomes remained approximately stable in the new study cohorts (Fig. 4). A small percentage of patients were lost during each cohort prior to reaching a full 5 years of follow up (Table S1 and Fig. S3). When we treated patients lost to follow up as having died rather than alive, we obtained similar model coefficients and predicted probabilities compared to those derived from treating those patients as alive at the end of the 5 years.

When we applied the original model to new cohorts, the C-Index ranged from 0.87 to 0.91 demonstrating high discriminative power (Fig. S4) similar to the C-Index of 0.89 in the original 1993 validation cohort (Fig. S5). Calibration was best in the 1993–1997 validation cohort with similar numbers of expected and observed deaths within sub-groups defined by risk scores (Table S4). Expected tended to be lower than observed numbers of deaths, with increasing differences with successive cohorts due to a bias towards over-optimistic estimates of predicted survival due to exclusion of transplanted patients (see limitations section of Discussion).

Table 3 shows results from logistic regression of death within 5-year follow-up on the prognostic score, using Eq. (2). Under a well calibrated model we should find an intercept of 0 (${\alpha }_{0}=0$) and a slope of 1 (${\alpha }_{1}=1$), as seen with the 1993–1997 validation cohort. The estimated slopes are close to 1 in all new cohorts, but the intercepts are greater than zero, indicating that original model predicted probabilities are too low. This also holds when the slope is fixed to be 1.

**Table 3:** Assessments of Model Calibration in New Cohorts from the US CFFPR, 1993–2016^*^.

Parameter	1993–1997 Validation Cohort, n = 5,810	New 1993–1997 Cohort, n = 9,941	New 1993–1998, n = 9,757	New 1999–2004, n = 13,073	New 2005–2010, n = 15,043	New 2011–2016, n = 17,635
Using Eq. (2)						
α_0_	0.046 (0.062)	0.33 (0.055)	0.62 (0.058)	0.74 (0.060)	0.59 (0.066)	0.64 (0.062)
α_1_	1.00 (0.034)	1.05 (0.027)	1.06 (0.027)	1.06 (0.025)	1.03 (0.025)	1.04 (0.024)
Using Eq. (2) with α_1_ fixed to be 1						
α_0_	0.029 (0.047)	0.26 (0.037)	0.52 (0.036)	0.61 (0.035)	0.53 (0.036)	0.54 (0.033)

*Results from using two strategies to assess model calibration are shown. See *Statistical Analysis* in **Methods** following Eq. (2). Results are shown as “Estimate (Standard Error).” Prognostic risk score is equivalent to log-odds of death within 5 years.

Findings from calibration assessments and similarities between coefficients in new models derived from new cohorts suggest that modifying the intercepts alone (Fig. 4) or both intercepts and slopes in the prediction model (${b}_{0}$ and ${\alpha }_{1}$, respectively) would improve the performance of the original model in the new cohorts. Modified intercepts alone, thus using α_0_ estimates with α_1_ set to 1 from Eq. (2), produced modified prognostic risk scores using Eq. (4) that improved model calibration in all new cohorts (Table S5). For the two most recent cohorts, modifying intercepts alone produced better calibration than using both new intercepts and slopes by using α_0_ and α_1_ estimates from Eq. (2) (Table S6).

Sensitivity analyses showed similar results using data with no attempts to correct potentially incorrect data with values within physiologic limits (for example accepting a physiologically plausible height of 165 cm without further testing vs deleting a recorded height of 1,650 cm) or using data after imputation of missing data for height, weight and FEV_1_. Results were similar whether using NHANES III^31^ or GLI^32^ equations to calculate FEV_1_%, although the choices of equations select somewhat different sets of study patients due to racial or ethnic differences. For example, patients of Asian race are excluded when using NHANES III while Hispanic ethnicity cannot be considered when using GLI to derive FEV_1_% because of the lack of applicable equations for race or ethnicity. Additional sensitivity analyses showed similar results when including patients who underwent lung transplantation during each cohort.

In summary, the prediction model has excellent discrimination in new cohorts. Model intercept modifications improve the calibration and accuracy of predicted probabilities of death within 5 years especially for recent cohorts. From Eq. (4), the modified intercept for the 2011 cohort is −1.38 which produces the most appropriate model for use today depending on the application (Original Model with Modified Intercept, Table S3).

## Discussion

We evaluated a previously published 5-year predicted survival model of CF and found that it remains a useful prediction model in updated cohorts. However, performance improved with adjustment of the model intercept to account for overall improvements in mortality rates over time. Coefficients for each included variable derived from new cohorts were similar (Fig. 3 and Table S3) showing that associations between demographic factors and measures of disease state remain largely unchanged despite 5-year survival improvements over time (Fig. 2). After intercept adjustment, the original 5-year prediction model has excellent calibration (Fig. 4 and Table S5), unchanged clinical implications and equally good discrimination for all new patient cohorts (Fig. S4 and Fig. S5). Five-year survival probabilities for CF improved because of slowing disease progression and shifts in distributions of most survival predictors (Fig. 1). These findings suggest that the original unmodified 5-year predicted survival model remains useful for stratifying individuals into expected survival groups for observational or interventional studies of CF^29^. Worksheets allow comparisons of survival predictions using original and modified models (Table S7). The model with modified intercept is more useful for applications where precise comparisons between individual predictions and outcomes are needed, for example, for investigation of the survival impact of lung transplantation^53,54^ in a setting of markedly improved survival with CF.

In the current study, all covariates included in the 5-year model except diabetes improved (Fig. 1), favoring better survival on average within each successive cohort. Some patients remain at every disease level, although the proportion of patients in the most severe states of disease continue to decrease (Table 2).

Unexpectedly, diabetes was more common at every age in each succeeding year of the CFFPR (Fig. 1e). By the end of the study period, the roughly 15% increase in CFRD was associated with an increase in mortality equal to approximately 20% of the observed decrease in mortality.

Multiple mechanisms cause CFRD including physical destruction of pancreatic β-islet cells from inflammation^55^, modifier gene influences^56,57^ and *CFTR* dysfunction itself^58,59^. Sustained increases in CFRD prevalence (Fig. 1E) may stem partially from competing influences of modestly improving CFRD detection^2,34^, mild phenotype frequency and weight-for-age *z*-score. However, improving survival may allow better observation of a direct effect of *CFTR* dysfunction suggesting that modulators of defective CFTR^25,26^ may modify CFRD pathogenesis and that CFRD biomarkers might be novel reporters to help guide use of these new agents. CFTR modulators may treat or prevent CFRD itself independently of lung disease.

The prediction model was fitted using logistic regression modeling of patients with complete data with and without methods to account for missing and incorrect data. Loss to follow up in the CFFPR was recently evaluated in a 2009–2013 cohort and involved less than 10% of patients^1^. We found similar occurrences of loss to follow up in our cohorts (Table S1, last row and Fig. S3). Sensitivity analyses using the four cohorts, 1993–1998, 1999–2004, 2005–2010 and 2011–2016 and treating patients who were lost to follow up as having died rather than as alive at the end of the 5-year period resulted in similar model coefficients and similar fits to the data with no effect on interpretation of our results. Independent evaluation found nearly complete clinical data for 2003–2009 and missingness of no more than 4.2% of death dates^60^. The high follow-up rates and low proportion of individuals lost to follow-up in the CFFPR data^1,60^ probably explain the lack of material differences between using uncorrected and corrected data and suggest that finding patients previously missing from the CFFPR may provide no further substantial changes in the prediction model.

The high degrees of model discrimination and calibration suggest that further improvements to the model by simply adding new variables to the model may be difficult. Addition of variables of high interest in the CF community include assessments of liver disease^61^, renal dysfunction^62^, arthropathy^54^ as well as severe and acute but unusual or rare events such as massive hemoptysis^63^ and pneumothorax^64^. Within the CFFPR, addition of any of these variables to the current logistic regression model eliminated large numbers of patients from assessment due to missingness of data, sometimes exceeding 50% of the patients included in the current study. This degree of missingness introduced severe bias to the analysis of survival (not shown).

Expansion of the model with novel variables is highly desirable but must avoid introduction of bias from exclusion of large numbers of patients with missing data. Inclusion of sufficient relevant events to assess variables in addition to those in our original multivariable model may be feasible using methods that incorporate longitudinal follow up over extended periods. Such a model could allow inclusion of sparsely collected or observed events such as pneumothorax and quantitatively relate their effects to those of more common factors such as low FEV_1_%. The present work, by demonstrating the stability of the 5-year predicted survival model using 5 cohorts collectively followed for over 24 consecutive years, provides the foundational work that supports the feasibility of such a longitudinal approach.

Some improvements to the prediction model might also be achieved by incorporating non-linear functions of continuous variables, *e.g*., age and FEV_1_%, and by treating prior pulmonary exacerbations as a categorical variable. Further, natural day-to-day fluctuations in FEV_1_%, which may be considered measurement error, could be assessed by other methods, such as joint modeling of the longitudinal FEV_1_% process and the survival process^65^.

Our study has limitations. The data are derived from non-randomly selected CFFPR participants and thus may include biases; however these should not be greater than for our prior work^30^ and should be reduced by intervening data cleaning^1^. We excluded patients too young for pulmonary function testing possibly leading to more pessimistic survivorship estimates: with current therapies, these patients tend to start and stay in the highest categories of health and therefore contribute infrequently to deaths in CF^2^. Higher variation in recording data of some variables, such as pancreatic sufficiency status (Fig. 1D) contributes to somewhat larger standard errors in variable estimates (Fig. 3); however, extensive data cleaning did not change our results or interpretations. Methods for recording pulmonary exacerbations changed in 2005 which might have changed the impact on survivorship; however, we are confident of the steady decreases in the numbers of pulmonary exacerbations before and after 2005 (Fig. 1H). We excluded lung transplantation recipients during each cohort period thus incorporating conditioning on a future event. However, reanalysis without transplant exclusions produced similar results with no impact on interpretations. (These results are insufficient, however, to allow comment on transplant survival effects.) The exclusion from the original 1993 cohort resulted in 5-year survival probabilities that were approximately 2% over-optimistic relative to observed survival, a bias that was not clinically meaningful in an analysis of lung transplantation survival outcomes^53^. Finally, many patients were eligible for and included in multiple study cohorts. This allowed assessments of the potential impacts of intervening data cleaning for cohorts beginning in 1993. For cohorts non-overlapping in time, inclusion of all eligible patients provided the most appropriate population for understanding survival during the specific study period.

We tested the published 5-year predicted survival model of CF derived from a 1993–1997 US cohort of patients using new cohorts because new treatments (including CFTR modulators for 2005–2010 and 2011–2016 cohorts) improved observed mortality rates. Results of re-derivation of 5-year survival models using updated cohorts were similar to original results and were stable through multiple sensitivity analyses even when patients lost to follow up were reclassified as being among the dead or including patients who received lung transplants during each cohort. The original model maintains good calibration and discrimination with new cohorts, especially in the most recent cohort with modified intercept alone, demonstrating the stability of both the model and the underlying disease processes of CF in the face of multiple effective therapies. CFRD is an increasingly important detection and treatment target with substantial potential for improving survival with CF. The 5-year predicted survival model in original and modified forms remains useful for disease categorization and individual prognosis, and the demonstrably stable effects of underlying variables provide a foundation for new models incorporating extended longitudinal follow up.

## Supplementary information

Supplementary figure S1.

Supplementary figure S2.

Supplementary figure S3.

Supplementary figure S4.

Supplementary figure S5.

Supplementary table S1.

Supplementary table S2.

Supplementary table S3.

Supplementary table S4.

Supplementary table S5.

Supplementary table S6.

Supplementary table S7.

Supplementary information.
