# Supplementary material for: Evaluation of a five-year predicted survival model for cystic fibrosis in later time periods
Source: Sci Rep. 2020 Apr 20;10:6602. doi: 10.1038/s41598-020-63590-8 (PMC7171119; doi:10.1038/s41598-020-63590-8)
Supplement: Supplementary file 6 — Supplementary table S1. [file 41598_2020_63590_MOESM6_ESM.docx]

**Table S1: Missingness of Data and Censoring for Modeling of 5-Year Predicted Survival by Cohorts from the US CFFPR, 1993-2016.**

|  | **Cohorts** | | | |
| --- | --- | --- | --- | --- |
|  | **1993-1998** | **1999-2004** | **2005-2010** | **2011-2016** |
| Patients in Registry | 17,771 | 20,326 | 22,407 | 26,391 |
| Patients older than 6 years | 13,594 | 16,567 | 18,667 | 21,915 |
| Patients Excluded for Lung Transplantation^*^ | 693 (5.10) | 1108 (6.69) | 1342 (7.19) | 1533 (7.00) |
| Heights^*^ | 472 (3.47) | 338 (2.04) | 156 (0.84) | 184 (0.84) |
| Heights^†^ | 1,203 (8.85) | 396 (2.39) | 236 (1.264) | 344 (1.57) |
| FEV_1_^*^ | 940 (6.92) | 796 (4.805) | 527 (2.823) | 493 (2.25) |
| FEV_1_^†^ | 1,979 (14.56) | 981 (5.92) | 656 (3.51) | 779 (3.56) |
| FEV_1_%^*^ | 1,164 (8.56) | 1025 (6.19) | 776 (4.16) | 946 (4.32) |
| FEV_1_%^†^ | 802 (5.90) | 742 (4.48) | 592 (3.17) | 673 (3.07) |
| Weight^*^ | 320 (2.35) | 231 (1.394) | 66 (0.3536) | 93 (0.4244) |
| Weight^†^ | 1376 (10.12) | 500 (3.018) | 249 (1.334) | 337 (1.538) |
| Weight-for-Age *z-*score^*^ | 323 (2.38) | 235 (1.42) | 78 (0.42) | 101 (0.46) |
| Weight-for-Age *z-*score^†^ | 322 (2.37) | 235 (1.42) | 78 (0.42) | 101 (0.46) |
| Diabetes status^*‡^ | 1 (0.007) | 1 (0.006) | 1 (0.005) | 245 (1.118) |
| Pancreatic Sufficiency status^*‡^ | 0 (0) | 0 (0) | 0 (0) | 0 (0) |
| Microbiology data^*^ | 1773 (13.04) | 1,358 (8.197) | 1,400 (7.5) | 1,099 (5.015) |
| Pulmonary Exacerbation data^*^ | 1,196 (8.80) | 1,118 (6.748) | 1,214 (6.503) | 1,436 (6.553) |
| 5-year predictions based on NHANES III FEV_1_%^*^ | 3,401 (25.02) | 4,442 (26.81) | 3,638 (19.49) | 3,583 (16.35) |
| 5-year predictions based on NHANES III FEV_1_%^§^ | 3,144 (23.13) | 4,094 (24.71) | 3,500 (18.75) | 3,403 (15.53) |
| 5-year predictions based on GLI FEV_1_%^*^ | 11,735 (86.32) | 16,513 (99.67) | 14,907 (79.86) | 16,666 (76.05) |
| 5-year predictions based on GLI FEV_1_%^§^ | 3,281 (24.14) | 4,300 (25.96) | 3,468 (18.58) | 3,291 (15.02) |
| Patients Censored as alive^**^ | 1566 (11.5) | 1800 (10.8) | 1639 (8.8) | 3078 (14) |

^*^ Number of Patients in CFFPR year excluded or missing value (percent of patients older than 6 years).

^†^ Number of patients in CFFPR year missing value after removal of incorrect values identified using generalized additive models methods (percent of patients older than 6 years). See Methods and Appendix Figure 1 for details of corrections.

^‡^ Diagnosis based on use of insulin for diabetes and pancreatic enzymes for pancreatic sufficiency. Pancreatic enzyme usage is required to enter data in the CFFPR thus missing data are never encountered.

^§^ Number of patients in CFFPR year missing value after correction of incorrect values identified using generalized additive models methods (percent of patients older than 6 years). See Methods and Appendix Figure S1 for details of corrections.

^**^ Number of patients with less than 5 years of follow up (percent of patients older than 6 years).
