# Supplementary material for: Evaluation of a five-year predicted survival model for cystic fibrosis in later time periods
Source: Sci Rep. 2020 Apr 20;10:6602. doi: 10.1038/s41598-020-63590-8 (PMC7171119; doi:10.1038/s41598-020-63590-8)
Supplement: Supplementary file 7 — Supplementary table S2. [file 41598_2020_63590_MOESM7_ESM.docx]

**Table S2. Associations with CF Related Diabetes in the US CFFPR, 1993-2016.**

|  | **Estimate** | **SE** | ***P*** |
| --- | --- | --- | --- |
| (Intercept) | -2.845 | 0.078 | <0.001 |
| FEV_1_%^*^ | -0.014 | 0.001 | <0.001 |
| Weight-for-age *z*-score | -0.228 | 0.015 | <0.001 |
| Age | 0.052 | 0.002 | <0.001 |
| CFFPR year | 0.090 | 0.002 | <0.001 |

^*^ All estimates were based on height, weight and FEV_1_ after corrections and imputations. FEV_1_% is based on NHANES III equations. Substitution of GLI derived values made minimal changes (<5 %) to the Estimates shown. See Methods in the main text.
