# Supplementary material for: Evaluation of a five-year predicted survival model for cystic fibrosis in later time periods
Source: Sci Rep. 2020 Apr 20;10:6602. doi: 10.1038/s41598-020-63590-8 (PMC7171119; doi:10.1038/s41598-020-63590-8)
Supplement: Supplementary file 8 — Supplementary table S3. [file 41598_2020_63590_MOESM8_ESM.docx]

**Table S3. Original, New and Modified Original Multivariable Logistic Regression Models of New Study Cohorts from the US CFFPR, 1993-2016.**

|  | **Coefficients^*^** | **Original Model**  **1993-1997 Development Cohort** | **New 1993-1997** | **New 1993-1998** | **New 1999-2004** | **New 2005-2010** | **New 2011-2016** | **Original Model with Modified Intercept** | **Original Model with Modified Intercept and Slope** |
| --- | --- | --- | --- | --- | --- | --- | --- | --- | --- |
| *b*_0_ | Intercept | -1.93 (0.28) | -1.07 (0.22) | -0.82 (0.22) | 0.42 (0.22)  *P =* 0.06 | -0.65 (0.22)  *P =* 0.003 | -0.58 (0.20)  *P =* 0.004 | -1.38 (0.28) | -1.37 (0.28) |
| *b*_1_ | Age | 0.028 (0.006) | 0.021 (0.004) | 0.018 (0.005) | 0.0077 (0.0042)  *P* = 0.07 | 0.020 (0.004) | 0.022 (0.003) | 0.028 (0.006) | 0.029 (0.06) |
| *b*_2_ | FEV_1_% | -0.038 (0.003) | -0.046 (0.002) | -0.044 (0.002) | -0.056 (0.002) | -0.044 (0.002) | -0.044 (0.002) | -0.038 (0.003) | -0.040 (0.06) |
| *b*_3_ | Weight-for-age *z-*score | -0.40 (0.053) | -0.42 (0.042) | -0.40 (0.042) | -0.23 (0.042) | -0.29 (0.0419) | -0.28 (0.038) | -0.40 (0.053) | -0.42 (0.082) |
| *b*_4_ | Sex | 0.23 (0.098) *P =* 0.018) | 0.0062 (0.076) *P =* 0.94 | 0.003 (0.076) *P =* 0.97 | -0.075 (0.075) *P =* 0.32 | -0.033 (0.074) *P =* 0.66 | -0.17 (0.069) *P =* 0.016 | 0.23 (0.098) *P =* 0.018 | 0.24 (0.12) *P =* 0.018 |
| *b*_5_ | *B. cepacia* complex (BCC) infection | 1.82 (0.30) | 1.3 (0.21) | 1.38 (0.22) | 1.53 (0.21) | 1.16 (0.23) | 0.88 (0.23) | 1.82 (0.30) | 1.90 (0.31) |
| *b*_6_ | Pancreatic Sufficiency | -0.45 (0.31)  *P* = 0.14 | -0.13 (0.19)  *P* = 0.48 | -0.17 (0.25)  *P* = 0.49 | -0.75 (0.25)  *P* = 0.002 | -0.36 (0.19)  *P* = 0.06 | -0.47 (0.16)  *P* = 0.003 | -0.45 (0.31)  *P* = 0.14 | -0.47 (0.32)  *P* = 0.14 |
| *b*_7_ | Methicillin Sensitive *S. aureus* | -0.22 (0.18)  *P* = 0.067 | -0.22 (0.09)  *P* = 0.016 | -0.23 (0.09)  *P* = 0.009 | 0.015 (0.08)  *P* = 0.86 | -0.29 (0.09)  *P* = 0.0018 | -0.41 (0.09) | -0.22 (0.12)  *P* = 0.07 | -0.22 (0.13)  *P* = 0.07 |
| *b*_8_ | Diabetes | 0.49 (0.15,)  *P* = 0.0013) | 0.68 (0.12) | 0.67 (0.11) | 0.51 (0.09) | 0.46 (0.08) | 0.42 (0.07) | 0.49 (0.15)  *P* = 0.001 | 0.51 (0.16)  *P* = 0.001 |
| *b*_9_ | Pulmonary Exacerbations in prior year | 0.46 (0.03) | 0.42 (0.025) | 0.47 (0.024) | 0.43 (0.022) | 0.50 (0.025) | 0.51 (0.023) | 0.46 (0.032) | 0.48 (0.070) |
| *b*_10_ | Interaction between BCC and Prior Exacerbations | -0.40 (0.118) | -0.15 (0.088)  *P* = 0.08 | -0.15 (0.10)  *P* = 0.11 | -0.21 (0.07)  *P* = 0.004 | -0.093 (0.10)  *P* = 0.37 | -0.030 (0.10)  *P* = 0.77 | -0.40 (0.12) | -0.42 (0.13) |

***^*^*** Results show estimates of log odds ratio (standard error). *P* values are < 0.001 except as noted.
