# Supplementary material for: Evaluation of a five-year predicted survival model for cystic fibrosis in later time periods
Source: Sci Rep. 2020 Apr 20;10:6602. doi: 10.1038/s41598-020-63590-8 (PMC7171119; doi:10.1038/s41598-020-63590-8)
Supplement: Supplementary file 9 — Supplementary table S4. [file 41598_2020_63590_MOESM9_ESM.docx]

**Table S4. Calibration of the 2001 5-Year Predicted Survival Model with New Cohorts from the US CFFPR, 1993-2016.**

| **Risk Score Sub-Group^*^** | **Deaths** | | | | | | | | | | | | | |
| --- | --- | --- | --- | --- | --- | --- | --- | --- | --- | --- | --- | --- | --- | --- |
|  | **1993-1997 Derivation Cohort, n = 5,820** | | **1993-1997 Validation Cohort, n = 5,810** | | **New 1993-1997 Cohort, n = 9,941** | | **New 1993-1998 Cohort, n = 9,757** | | **New 1999-2004 Cohort, n = 13,073** | | **New 2005-2010 Cohort, n = 15,043** | | **New 2011-2016 Cohort, n = 17,635** | |
|  | **Exp^†^** | **Obs^†^** | **Exp^†^** | **Obs^†^** | **Exp^†^** | **Obs^†^** | **Exp^†^** | **Obs^†^** | **Exp^†^** | **Obs^†^** | **Exp^†^** | **Obs^†^** | **Exp^†^** | **Obs^†^** |
| 1 | 1 | 3 | 1 | 0 | 3 | 8 | 2 | 8 | 2 | 5 | 2 | 5 | 2 | 2 |
| 2 | 3 | 3 | 3 | 8 | 6 | 9 | 5 | 12 | 4 | 5 | 4 | 13 | 4 | 10 |
| 3 | 6 | 10 | 6 | 5 | 9 | 15 | 8 | 14 | 6 | 6 | 7 | 12 | 7 | 13 |
| 4 | 10 | 7 | 9 | 6 | 14 | 16 | 12 | 14 | 10 | 13 | 10 | 13 | 10 | 15 |
| 5 | 16 | 13 | 16 | 18 | 23 | 21 | 19 | 29 | 14 | 19 | 15 | 20 | 16 | 28 |
| 6 | 28 | 28 | 29 | 26 | 38 | 36 | 33 | 41 | 23 | 28 | 22 | 26 | 25 | 24 |
| 7 | 49 | 39 | 53 | 49 | 66 | 62 | 57 | 80 | 39 | 60 | 37 | 56 | 42 | 71 |
| 8 | 88 | 90 | 96 | 102 | 117 | 141 | 103 | 147 | 76 | 139 | 67 | 117 | 77 | 109 |
| 9 | 168 | 177 | 180 | 190 | 231 | 307 | 206 | 319 | 173 | 308 | 142 | 224 | 162 | 296 |
| 10 | 343 | 343 | 353 | 355 | 527 | 598 | 491 | 627 | 534 | 721 | 464 | 643 | 524 | 721 |
| Totals | 712 | 713 | 746 | 759 | 1034 | 1213 | 936 | 1291 | 881 | 1304 | 770 | 1129 | 869 | 1289 |
| Hosmer-Lemeshow Test, χ^2^ (*P*) | 9.21 (0.32) | | 11.7 (0.17) | | 76.6 (<0.001) | | 226 (<0.001) | | 310 (<0.001) | | 231 (< 0.001) | | 286 (<0.001) | |

**^*^** Patients in each cohort were divided into tenths for Hosmer-Lemeshow testing. The range of predictions within the sub-groups differed slightly between cohorts.

^†^ Abbreviations: Exp=Expected number of deaths within 5 years rounded to the nearest integer. Obs=Number of deaths observed within 5 years for the cohort.
