# Supplementary material for: Evaluation of a five-year predicted survival model for cystic fibrosis in later time periods
Source: Sci Rep. 2020 Apr 20;10:6602. doi: 10.1038/s41598-020-63590-8 (PMC7171119; doi:10.1038/s41598-020-63590-8)
Supplement: Supplementary file 10 — Supplementary table S5. [file 41598_2020_63590_MOESM10_ESM.docx]

**Table S5. Calibration of the 2001 5-Year Predicted Survival Model with Modified Intercepts with New Cohorts from the US CFFPR, 1993-2016.**

| **Risk Score Sub-Group^*^** | **Deaths** | | | | | | | | | | | | | |
| --- | --- | --- | --- | --- | --- | --- | --- | --- | --- | --- | --- | --- | --- | --- |
|  | **1993-1997 Derivation Cohort, n = 5,820** | | **1993-1997 Validation Cohort, n = 5,810** | | **New 1993-1997 Cohort, n = 9941** | | **New 1993-1998 Cohort, n = 9757** | | **New 1999-2004 Cohort, n = 13073** | | **New 2005-2010 Cohort, n = 15043** | | **New 2011-2016 Cohort, n = 17635** | |
|  | **Exp^†^** | **Obs^†^** | **Exp^†^** | **Obs^†^** | **Exp^†^** | **Obs^†^** | **Exp^†^** | **Obs^†^** | **Exp^†^** | **Obs^†^** | **Exp^†^** | **Obs^†^** | **Exp^†^** | **Obs^†^** |
| 1 | 1 | 3 | 1 | 0 | 2 | 2 | 2 | 2 | 2 | 4 | 3 | 2 | 3 | 2 |
| 2 | 3 | 3 | 3 | 8 | 6 | 14 | 5 | 12 | 5 | 6 | 6 | 10 | 6 | 6 |
| 3 | 6 | 10 | 6 | 5 | 9 | 11 | 9 | 11 | 9 | 7 | 9 | 9 | 10 | 12 |
| 4 | 10 | 7 | 9 | 6 | 15 | 15 | 15 | 16 | 14 | 8 | 14 | 13 | 16 | 20 |
| 5 | 16 | 13 | 16 | 18 | 25 | 20 | 26 | 22 | 22 | 19 | 22 | 26 | 24 | 22 |
| 6 | 28 | 28 | 29 | 26 | 42 | 42 | 45 | 40 | 37 | 30 | 34 | 22 | 39 | 38 |
| 7 | 49 | 39 | 54 | 49 | 76 | 57 | 82 | 84 | 66 | 64 | 58 | 63 | 67 | 63 |
| 8 | 88 | 90 | 97 | 102 | 138 | 132 | 152 | 130 | 131 | 144 | 106 | 115 | 127 | 119 |
| 9 | 168 | 177 | 184 | 190 | 270 | 292 | 302 | 320 | 295 | 303 | 227 | 227 | 259 | 282 |
| 10 | 343 | 343 | 359 | 355 | 575 | 573 | 614 | 616 | 730 | 726 | 631 | 623 | 716 | 702 |
| Totals | 712 | 713 | 758 | 759 | 1158 | 1158 | 1252 | 1253 | 1311 | 1311 | 1110 | 1110 | 1267 | 1266 |
| Hosmer-Lemeshow Test, χ^2^ (*P*) | 9.21 (0.32) | | 10.996 (0.202) | | 23.95 (0.002) | | 21.786 (0.005) | | 18.042 (0.021) | | 10.18 (0.253) | | 6.558 (0.585) | |

^*^ Patients in each cohort were divided into tenths for Hosmer-Lemeshow testing. The range of predictions within the sub-groups differed slightly between cohorts.

^†^ Abbreviations: Exp=Expected number of deaths within 5 years rounded to the nearest integer. Obs=Number of deaths observed within 5 years for the cohort.
