# Supplementary material for: Evaluation of a five-year predicted survival model for cystic fibrosis in later time periods
Source: Sci Rep. 2020 Apr 20;10:6602. doi: 10.1038/s41598-020-63590-8 (PMC7171119; doi:10.1038/s41598-020-63590-8)
Supplement: Supplementary file 11 — Supplementary table S6. [file 41598_2020_63590_MOESM11_ESM.docx]

**Table S6. Calibration of the 2001 5-Year Predicted Survival Model with Modified Intercepts and Slopes with New Cohorts from the US CFFPR, 1993-2016.**

| **Deciles^*^** | **Deaths** | | | | | | | | | | | | | |
| --- | --- | --- | --- | --- | --- | --- | --- | --- | --- | --- | --- | --- | --- | --- |
|  | **1993-1997 Derivation Cohort, n = 5,820** | | **1993-1997 Validation Cohort, n = 5,810** | | **New 1993-1997 Cohort, n = 9941** | | **New 1993-1998 Cohort, n = 9757** | | **New 1999-2004 Cohort, n = 13073** | | **New 2005-2010 Cohort, n = 15043** | | **New 2011-2016 Cohort, n = 17635** | |
|  | **Exp**^†^ | **Obs**^†^ | **Exp**^†^ | **Obs**^†^ | **Exp**^†^ | **Obs**^†^ | **Exp**^†^ | **Obs**^†^ | **Exp**^†^ | **Obs**^†^ | **Exp**^†^ | **Obs**^†^ | **Exp**^†^ | **Obs**^†^ |
| 1 | 1 | 3 | 1 | 0 | 3 | 8 | 3 | 8 | 3 | 5 | 3 | 5 | 3 | 2 |
| 2 | 3 | 3 | 3 | 8 | 6 | 9 | 6 | 12 | 6 | 5 | 7 | 13 | 6 | 10 |
| 3 | 6 | 10 | 6 | 5 | 10 | 15 | 11 | 14 | 9 | 6 | 10 | 12 | 10 | 13 |
| 4 | 10 | 7 | 9 | 6 | 16 | 16 | 17 | 14 | 15 | 13 | 15 | 13 | 16 | 15 |
| 5 | 16 | 13 | 16 | 18 | 26 | 21 | 29 | 29 | 22 | 19 | 23 | 20 | 24 | 28 |
| 6 | 28 | 28 | 29 | 26 | 45 | 36 | 49 | 41 | 36 | 28 | 36 | 26 | 39 | 24 |
| 7 | 49 | 39 | 54 | 49 | 80 | 62 | 87 | 80 | 64 | 60 | 60 | 56 | 67 | 71 |
| 8 | 88 | 90 | 97 | 102 | 144 | 141 | 158 | 147 | 126 | 139 | 109 | 117 | 125 | 109 |
| 9 | 168 | 177 | 184 | 190 | 282 | 307 | 307 | 319 | 286 | 308 | 227 | 224 | 261 | 296 |
| 10 | 343 | 343 | 359 | 355 | 600 | 598 | 623 | 627 | 737 | 721 | 639 | 643 | 739 | 721 |
| Totals | 712 | 713 | 758 | 759 | 1212 | 1213 | 1290 | 1291 | 1304 | 1304 | 1129 | 1129 | 1290 | 1289 |
| Hosmer-Lemeshow Test,  χ-square (*P*-value) | 9.21 (0.32) | | 11.43 (0.18) | | 25.2 (0.001) | | 19.3 (0.013) | | 10.6 (0.22) | | 12.2 (0.14) | | 18.2 (0.02) | |

^*^ Patients in each cohort were divided into deciles for Hosmer-Lemeshow testing. The range of predictions within deciles differed slightly between cohorts using the ResourceSelection package in R.

^†^ Abbreviations: Exp=Expected deaths rounded to the nearest integer. Obs=Number of deaths observed by the end of the observation period for the cohort.
