# Supplementary material for: Evaluation of a five-year predicted survival model for cystic fibrosis in later time periods
Source: Sci Rep. 2020 Apr 20;10:6602. doi: 10.1038/s41598-020-63590-8 (PMC7171119; doi:10.1038/s41598-020-63590-8)
Supplement: Supplementary file 12 — Supplementary table S7. [file 41598_2020_63590_MOESM12_ESM.docx]

**Table S7a. Worksheet 1.** **Calculation of weight-for-age *z* score.^*^**

| 1 | **Enter** the patient’s weight on line 1. | 1 |  |
| --- | --- | --- | --- |
| 2 | For the appropriate gender and approximate age (use 18 if age > 18),  **enter** median weight from table on line 2. | 2 |  |
| 3 | **Subtract** line 2 from line 1 and **enter** on line 3. | 3 |  |
| 4 | *—If* line 3 is zero, **skip** to line 5 and **enter** zero.  *—If* line 3 is less than zero, **enter** lower SD for age and gender on line 4.  *—If* line 3 is greater than zero, **enter** upper SD for age and gender on line 4. | 4 |  |
| 5 | *z* score: **divide** line 3 by line 4 and **enter** result on line 5. | 5 |  |

|  | Males | | | |  | Females | | | |  |
| --- | --- | --- | --- | --- | --- | --- | --- | --- | --- | --- |
|  | Age (years) | Median Wt (kg)^†^ | Upper SD^‡^ | Lower SD^‡^ |  | Age (years) | Median Wt (kg)^†^ | Upper SD^‡^ | Lower SD^‡^ |  |
|  | 5.5  6  6.5  7  7.5  8  8.5  9  9.5  10  10.5  11  11.5  12  12.5  13  13.5  14  14.5  15  15.5  16  16.5  17  17.5  18 | 19.67  20.69  21.74  22.85  24.03  25.30  26.66  28.13  29.73  31.44  33.30  35.30  37.46  39.78  42.27  44.95  47.81  50.77  53.76  56.71  59.51  62.10  64.39  66.31  67.78  68.88 | 3.04  3.45  3.91  4.43  5.30  5.62  6.28  6.98  7.70  8.43  9.15  9.86  10.53  11.16  11.74  12.24  12.62  13.02  13.35  13.66  13.99  14.34  14.76  15.24  15.82  16.39 | 2.18  2.29  2.41  2.57  2.75  2.99  3.26  3.59  3.95  4.34  4.75  5.18  5.62  6.05  6.48  6.90  7.29  7.65  7.99  8.29  8.54  8.76  8.92  9.03  9.08  9.09 |  | 5.5  6  6.5  7  7.5  8  8.5  9  9.5  10  10.5  11  11.5  12  12.5  13  13.5  14  14.5  15  15.5  16  16.5  17  17.5  18 | 18.56  19.52  20.61  21.84  23.26  24.84  26.58  28.46  30.45  32.55  34.72  36.95  39.23  41.53  43.84  46.10  48.26  50.28  52.10  53.68  54.96  55.89  56.44  56.69  56.71  56.62 | 3.38  3.80  4.26  4.78  5.37  6.02  6.71  7.43  8.17  8.91  9.66  10.40  11.09  11.76  12.37  12.93  13.44  13.90  14.32  14.70  15.02  15.30  15.54  15.71  15.80  15.76 | 1.99  2.12  2.29  2.52  2.83  3.18  3.60  4.05  4.51  4.99  5.47  5.92  6.34  6.71  7.04  7.29  7.49  7.63  7.71  7.74  7.71  7.61  7.46  7.29  7.09  6.93 |  |

^*^ Reproduced from Liou TG, Adler FR, Fitzsimmons SC, et al. Predictive 5-year survivorship model of cystic fibrosis. *Am J Epidemiol*. 2001;153(4):345–52. This table is no longer available at the original website.

For weights above the age and sex specific median,

*z score* = (*patient weight* - *median weight*)/*upper SD*.

For weights below the age and sex specific median,

*z score* = (*patient weight* - *median weight*)/*lower SD*.

Use of two equations recognizes that weight-for-age is not normally distributed (*From*: Lai H-C, Kosorok MR, Sondel SA, et al. Growth status in children with cystic fibrosis based on the National Cystic Fibrosis Patient Registry data: Evaluation of various criteria used to identify malnutrition. J Pediatr 1998;132:478-85, *and* Dibley MJ, Goldsby JB, Staehling NW, et al. Development of normalized curves for the international growth reference: historical and technical considerations. Am J Clin Nutr 1987;46:736-48).

^†^ *From*: Hamill PVV, Drizd TA, Johnson CL, et al. Physical growth: National Center for Health Statistics percentiles. Am J Clin Nutr 1979;32:607-29.

^‡^ The upper and lower standard deviations for each sex and age are calculated:

*upper SD* = (*95^th^ percentile weight* - *median weight*)/*1.64*

and

*lower SD* = (*median weight* - *5^th^ percentile weight*)/*1.64*

where 1.64 is the number of standard deviations for a cumulative probability of 5% and 95% for a normal distribution (*From*: Lai H-C, Kosorok MR, Sondel SA, et al. Growth status in children with cystic fibrosis based on the National Cystic Fibrosis Patient Registry data: Evaluation of various criteria used to identify malnutrition. J Pediatr 1998;132:478-85 *and* Dibley MJ, Goldsby JB, Staehling NW, et al. Development of normalized curves for the international growth reference: historical and technical considerations. Am J Clin Nutr 1987;46:736-48).

**Table S7b. Modified Worksheet 2 for the Five Year Survival Prediction Model with Intercept or Intercept and Slope modifications derived from the US CFFPR, 1993-2016.^*^**

| 1a | Baseline Score (Original Model). |  |  | 1a | 50 |
| --- | --- | --- | --- | --- | --- |
| 1b | Baseline Score (Model with Modified Intercept). |  |  | 1b | 36 |
| 1c | Baseline Score (Model with Modified Intercept and Slope). |  |  | 1c | 34 |
| 2 | Patient’s age: _____ **Multiply** by 0.7 and **Enter** on line 2. | 2 |  |  |  |
| 3 | *If* patient is female, **enter** 6 on line 3. | 3 |  |  |  |
| 4 | *If* patient has diabetes, **enter** 13 on line 4. | 4 |  |  |  |
| 5 | *If* patient has *B. cepacia* complex infection, **enter** 48 on line 5. | 5 |  |  |  |
| 6 | Number of pulmonary exacerbations in last year: _____ (Maximum = 5)  –*If* patient **has** *B. cepacia*, **multiply** by 2 and **enter** on line 6.  –*If* patient does **not have** *B. cepacia*, **multiply** by 12 and **enter** on line 6. | 6 |  |  |  |
| 7 | **Add** lines 2, 3, 4, 5 and 6 and **enter** on line 7. |  |  | 7 |  |
| 8 | **Subtract** line 7 from line 1a, b or c depending on model chosen, and **enter** on line 8. (Result may be negative). |  |  | 8 |  |
| 9 | Patient’s weight-for-age *z* score (from line 5, worksheet 1^†^): _____  –**Multiply** by 10 and **enter** on line 9. (Number may be negative.) | 9 |  |  |  |
| 10 | *If* patient has pancreatic sufficiency, **enter** 12 on line 10. | 10 |  |  |  |
| 11 | *If* patient is infected with *S. aureus*, **enter** 6 on line 11. | 11 |  |  |  |
| 12 | **Enter** the patient’s FEV_1_% on line 12.  (Normalized using either NHANES III or GLI equations) | 12 |  |  |  |
| 13 | **Add** lines 9 through 12 and **enter** result on line 13 |  |  | 13 |  |
| 14 | Raw score: **Add** lines 8 and 13 and **enter** on line 14 |  |  | 14 |  |
| 15 | **Use** the raw score and the table below to estimate prediction of 5 year survival and **enter** result on line 15. |  |  | 15 |  |

| **Raw Score** | -77 | -58 | -46 | -36 | -29 | -22 | -16 | -11 | -5 | 0 | 5 | 11 | 16 | 22 | 29 | 36 | 46 | 58 | 77 |
| --- | --- | --- | --- | --- | --- | --- | --- | --- | --- | --- | --- | --- | --- | --- | --- | --- | --- | --- | --- |
| **Prediction of 5 Year**  **Survival (percent)** | 5 | 10 | 15 | 20 | 25 | 30 | 35 | 40 | 45 | 50 | 55 | 60 | 65 | 70 | 75 | 80 | 85 | 90 | 95 |

**^*^** Reproduced and modified from Liou TG, Adler FR, Fitzsimmons SC, et al. Predictive 5-year survivorship model of cystic fibrosis. *Am J Epidemiol*. 2001;153(4):345–52. Worksheet 2 differs from the original with addition of lines 1b and 1c. The original worksheet is no longer available at the original website.

^†^ *From*: Hamill PVV, Drizd TA, Johnson CL, et al. Physical growth: National Center for Health Statistics percentiles. Am J Clin Nutr 1979;32:607-29.
