# Supplementary material for: Evaluation of a five-year predicted survival model for cystic fibrosis in later time periods
Source: Sci Rep. 2020 Apr 20;10:6602. doi: 10.1038/s41598-020-63590-8 (PMC7171119; doi:10.1038/s41598-020-63590-8)
Supplement: Supplementary file 13 — Supplementary information. [file 41598_2020_63590_MOESM13_ESM.docx]

**Supplemental Online Materials**

**Evaluation of a five-year predicted survival model for cystic fibrosis in later time periods**

Theodore G. Liou

Christiana Kartsonaki

Ruth H. Keogh

Frederick R. Adler

**Appendix Figure Legends**

**Figure S1. Identification and Correction using Generalized Additive Models (GAM) of Incorrect Height, Weight and FEV1 Values from the US CFFPR, 1993-2016.** We show examples of using recorded (**a**) Height, (**b**) Weight and (**c**) FEV_1_ (including a transplant-related increase at age 42) patient-by-patient with age as the independent variable (black circles) to identify individual values that had high absolute value *z*-scores for residuals (Utah red dots) for replacement (green dots and arrows) with GAM fitted values (orange). Data shown are based on patterns seen in the CFFPR but do not match any specific patients.

**Figure S2. Histograms of Prognostic Risk Scores and Predicted Probability of Death for Each Study Cohort from the US CFFPR, 1993-2016.** The distribution of prognostic risk scores and predicted probabilities of death (insets) have slightly changed over time from the (**a**) original 1993-1997 study cohort through later study cohorts (**b-f**).

**Figure S3. Histograms of Patients Lost to Followup during Each Study Cohort from the US CFFPR, 1993-2016.** The distribution of patients lost to follow up during 5-year follow up is shown for 6 month intervals for each cohort. The largest bars in each plot show the patients who completed follow up or had recorded deaths during each cohort period.

**Figure S4. Receiver Operator Curves for application of the Original 5-Year Predicted Survival Model to Each Study Cohort from the US CFFPR, 1993-2016.** Plots of sensitivity and specificity show discrimination performance of the original 5-year predicted survival model when applied to study cohorts: (**a**) Original 1993-1997, (**b**) New 1993-1997, (**c**) 1993-1998, (**d**) 1999-2004, (**e**) 2005-2010, (**f**) 2011-2016. Colors denote potential cutoff values identifying sensitivity and specificity for any chosen prediction value. Insets show density plots of bootstrapped determinations of area under the curve or C-Index for each receiver operator curve.

**Figure S5. Superimposed Receiver Operator Curves for Applications of the Original 5-Year Predicted Survival Model to Each Study Cohort from the US CFFPR, 1993-2016.** A plot of sensitivity and specificity shows discrimination performance of the original 5-year predicted survival model when applied to study cohorts allowing direct comparison with the performance of the original validation cohort from 1993-1997. Colors denote potential cutoff values identifying sensitivity and specificity for any chosen prediction value.
